# The Cystic Fibrosis Transmembrane Conductance Regulator (CFTR) Uses its C-Terminus to Regulate the A2B Adenosine Receptor

**DOI:** 10.1038/srep27390

**Published:** 2016-06-09

**Authors:** Michael J. Watson, Shernita L. Lee, Abigail J. Marklew, Rodney C. Gilmore, Martina Gentzsch, Maria F. Sassano, Michael A. Gray, Robert Tarran

**Affiliations:** 1 Cystic Fibrosis Center/Marsico Lung Institute, 125 Mason Farm Road, The University of North Carolina, Chapel Hill, NC, 27599-7248, USA; 2 Institute for Cell and Molecular Biosciences, Newcastle University, Newcastle-upon-Tyne, Northumberland, UK; 3 Cell Biology and Physiology, 111 Mason Farm Rd, The University of North Carolina, Chapel Hill, NC, 27599-7248, USA.

## Abstract

CFTR is an apical membrane anion channel that regulates fluid homeostasis in many organs including the airways, colon, pancreas and sweat glands. In cystic fibrosis, CFTR dysfunction causes significant morbidity/mortality. Whilst CFTR’s function as an ion channel has been well described, its ability to regulate other proteins is less understood. We have previously shown that plasma membrane CFTR increases the surface density of the adenosine 2B receptor (A2BR), but not of the β2 adrenergic receptor (β2AR), leading to an enhanced, adenosine-induced cAMP response in the presence of CFTR. In this study, we have found that the C-terminal PDZ-domain of both A2BR and CFTR were crucial for this interaction, and that replacing the C-terminus of A2BR with that of β2AR removed this CFTR-dependency. This observation extended to intact epithelia and disruption of the actin cytoskeleton prevented A2BR-induced but not β2AR-induced airway surface liquid (ASL) secretion. We also found that CFTR expression altered the organization of the actin cytoskeleton and PDZ-binding proteins in both HEK293T cells and in well-differentiated human bronchial epithelia. Furthermore, removal of CFTR’s PDZ binding motif (ΔTRL) prevented actin rearrangement, suggesting that CFTR insertion in the plasma membrane results in local reorganization of actin, PDZ binding proteins and certain GPCRs.

Cystic fibrosis (CF) is a multi-organ disease caused by genetic mutations in CFTR, a cAMP-regulated anion channel^1^, leading to a reduction or ablation in apical membrane CFTR channel activity that is especially visible in the lungs, pancreas, GI tract, sweat glands and reproductive systems^2^. In CF airways, lack of functional CFTR causes airway surface liquid (ASL) volume depletion/mucus dehydration, that leads to mucus plugging and chronic infection/inflammation that ultimately destroy the lungs^3^. Extracellular adenosine signals through purinergic receptors including the G_s_-linked A2B receptor (A2BR) which stimulates adenylate cyclase to raise cAMP and activate protein kinase A^4^,^5^. Regulation of CFTR by the A2BR is vital for the defense of mucosal surfaces^6^,^7^,^8^. For example, adenosine acts as a reporter molecule in the ASL whose concentration and dilution serve to modulate CFTR activity via stimulation of A2BR and hence, to maintain sufficient ASL volume to preserve mucus hydration^9^. A failure of this system, due to the lack of the effector (i.e. CFTR), contributes to the ASL depletion/mucus stasis seen in CF lung disease that leads to mucus stasis and chronic infections^10^,^11^.

CFTR is indirectly anchored to the actin cytoskeleton through filamin, NHERF1, NHERF2 and syntaxin^12^,^13^,^14^, while A2BR has been shown to bind NHERF2^15^. CFTR regulates other apical proteins including ENaC, KIR 1.1 and SLC26A9, through PDZ-binding via ezrin, NHERF1 and NHERF2, as well as through direct binding to CFTR^14^,^16^,^17^,^18^. These interactions are robust, and when CFTR-containing patches are excised from the plasma membrane, A2BR and signaling proteins such as G_s_ are also excised, allowing A2BR to regulate CFTR^19^. Similarly, Naren *et al*. demonstrated that CFTR and the β2 adrenergic receptor (β2AR) exist in an ordered macromolecular complex^20^. There is no *a priori* reason why CFTR should directly interact with its upstream G-protein-coupled receptor (GPCR) since several intermediary proteins, as well as soluble secondary messengers, lie between them in the signal transduction pathway. However, such a macromolecular complex may aid in increasing signal transduction specificity and efficiency in the plasma membrane.

We have previously shown that CFTR enhances A2BR protein expression in the plasma membrane of both non-polarized cells (BHK^CFTR^ and HEK293T cells) and well-differentiated, polarized human bronchial epithelial cultures (HBECs)^21^. Lack of CFTR-dependent regulation of A2BR leads to decreased cAMP production, ciliary beat frequency, and IL-8 secretion in response to adenosine in CF HBECs. CFTR-A2BR interactions also increase upon adenosine-exposure. For example, Forster Resonance Energy Transfer (FRET) between CFTR and A2BR acutely increases ~4 fold upon adenosine stimulation^21^. This interaction is specific for CFTR-A2BR and despite existing in a macromolecular complex^20^, FRET levels between CFTR and the β2AR do not alter upon stimulation with isoproterenol^21^. Despite having clear effects on CFTR function, little is known regarding how CFTR and A2BR interact. Recently, Monterisi *et al*. have reported (i) that CFTR affects actin and cAMP distribution in an airway cell line and that disruption of the actin cytoskeleton with latrunculin abolishes cAMP-dependent Cl^−^ efflux^22^. Since CFTR and A2BR are both connected to the actin cytoskeleton via their PDZ motifs, we have tested the hypothesis that agonist-induced CFTR-A2BR clustering and actin organization were dependent on C-terminal PDZ interactions.

## Results

### The organization of actin and other scaffolding proteins is disrupted in CF airway epithelia

**Figure 1:**
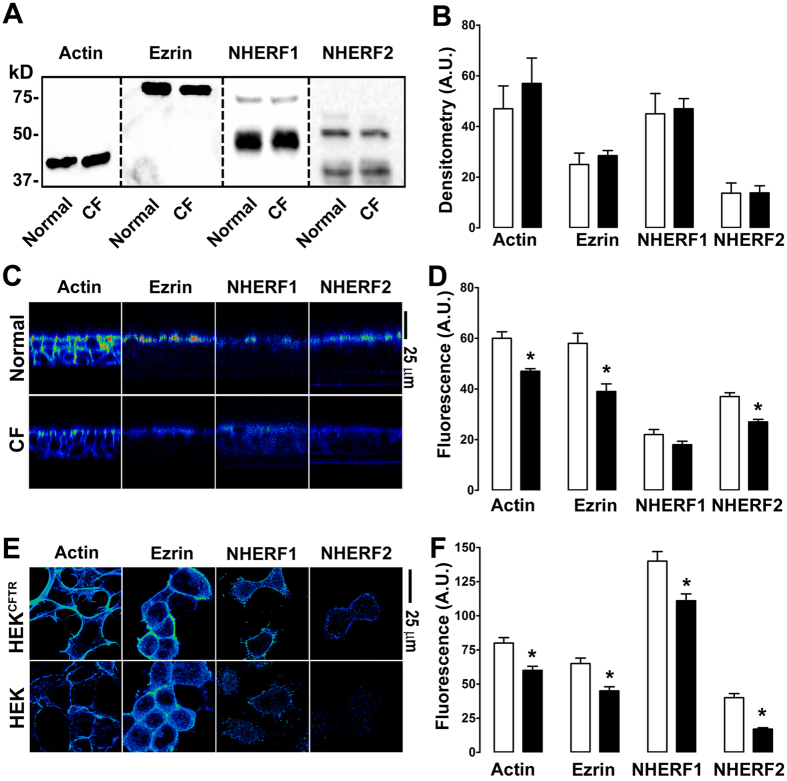
CFTR affects the near-membrane organization of scaffolding proteins. Open bars, normal or CFTR-expressed; closed bars, CF or null as appropriate. (**A**) Typical Western blot of actin, ezrin, NHERF1 and NHERF2 in normal and CF HBECs. (**B**) Mean densitometry data for apical and sub-apical actin, ezrin and NHERF1/2. (**C**) Representative pseudocolored confocal micrographs taken in X-Z mode showing F-actin, ezrin, NHERF1 and NHERF2 staining in normal and CF HBECs. (**D**) Mean fluorescent intensities taken from C. (**E**) Representative pseudocoloured confocal micrographs taken in X-Y mode showing F-actin, ezrin, NHERF1 and NHERF2 localization in HEK cells and HEK cells stably expressing CFTR (HEK^CFTR^). (**F**) Mean near plasma membrane fluorescence image intensities. All data are representative from images acquired on three separate occasions. HBECs were provided from 3 normal and 3 CF (ΔF508/ΔF508) donors. *p < 0.05 different ± CFTR expression.

It has previously been shown that CFTR alters actin expression in an airway epithelial cell line (16HBE)^22^. We tested the hypothesis that this phenomenon was preserved in primary airway epithelial cultures from normal vs. CF donors. We also hypothesized that CFTR could also influence the localization of scaffolding/PDZ-binding proteins. To test these hypotheses, we searched in primary HBECs for altered actin, ezrin, NHERF1 and NHERF2 expression in the presence of wild-type CFTR vs. ΔF508 CFTR that does not traffic to the plasma membrane. CFTR had no effect on total actin, ezrin, NHERF1 and NHERF2 protein levels, as measured by Western blotting in normal vs. ΔF508 CFTR homozygous CF HBECs (Fig. 1A,B). However, expression of wild-type CFTR induced a significant reorganization of actin, ezrin and NHERF2 with more protein being visible at the apical plasma membrane, an effect that was diminished in CF/ΔF508-CFTR HBECs (Fig. 1C,D). A similar difference in the organization of these proteins could be detected in HEK293T cells expressing wild-type CFTR (Fig. 1E,F).

**Figure 2:**
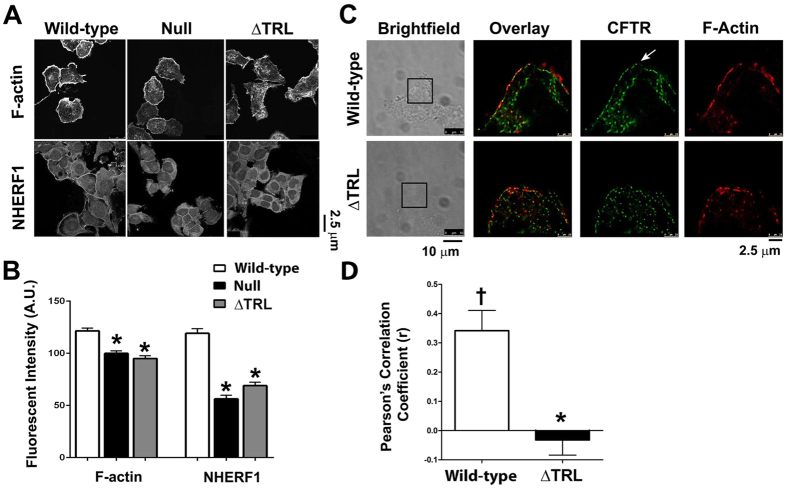
The C-Terminus of CFTR is required for focusing the sub-apical cytoskeleton. (**A**) Typical confocal micrographs taken in X-Y mode of F-actin and NHERF1 in HEK293T cells expressing GFP-tagged wild-type or ΔTRL CFTR, or non-transfected (null). Scale bar is 25 μm. (**B**) Mean fluorescent intensities taken from confocal micrographs in A. All n = 15. (**C**) Typical super resolution images of wild-type and ΔTRL CFTR (green) expressed in HEK293T cells and counter-stained for F-actin using phalloidin-647 (red). Each pixel is 30 nm^2^ and the scale bar is 2.5 μm. Arrow denotes plasma membrane wild-type CFTR. All data are representative from images acquired on three separate occasions. The corresponding brightfield images are also shown and the regions imaged in super resolution mode are boxed. (**D**) Pearson’s correlation coefficient (r) for colocalization between wild-type or ΔTRL CFTR and F-actin, based on images shown in C. *p < 0.05 different ± CFTR expression. ^†^p < 0.05 different to 0.

Since CFTR has previously been shown to interact with actin via its C-terminal PDZ-binding motif, we next tested whether deletion of this motif (CFTR^ΔTRL^) affected actin and NHERF1 organization. Indeed, CFTR^ΔTRL^ did not affect either actin or NHERF1 and cells expressing this construct were similar to null HEK293T cells that did not express CFTR (Fig. 2A,B). Further investigation using super resolution microscopy indicated that CFTR and actin could be detected in a discrete region at the cell periphery of HEK293T cells that was clearly demarked from intracellular CFTR/actin (Fig. 2C). In contrast, both CFTR^ΔTRL^ and actin were visible in a more diffused pattern throughout the cell and it was hard to differentiate between plasma membrane vs. intracellular CFTR (Fig. 2C). The Pearson’s coefficient (r) for wild-type CFTR and actin (phalloidin) was ~0.35, suggesting that the two proteins significantly colocalized (Fig. 2D). In contrast, r was significantly reduced to <0 for CFTR^ΔTRL^, which indicated that colocalization no longer occurred (Fig. 2D).

**Figure 3:**
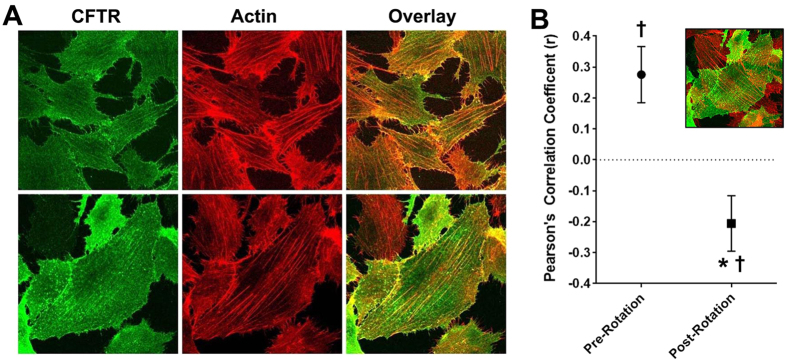
Surface CFTR co-localizes with actin. (**A**) HA-CFTR (green) was labelled with anti-HA primary and Alexa488 goat secondary antibody in BHK^CFTR^ cells. The actin cytoskeleton (red) was labelled with Phalloidin 568. Images were taken sequentially to ensure full spectral separation and are representative of three independent experiments. (**B**) Mean Pearson’s correlation coefficient (r) for CFTR and actin aligned as per panel a (pre-rotation, ●) and after rotating the actin images by 90° (post-rotation, ■). Both n = 16. Inset shows actin with 90° rotation. * denotes p < 0.05 different between pre and post-rotation. ^†^p < 0.05 different to 0.

We next tested whether surface CFTR could be localized near actin. Accordingly, we visualized surface CFTR in BHK^CFTR^ cells without staining intracellular CFTR (Fig. 3). It is important to note that for these experiments, surface CFTR was first labelled, and then cells were permeabilized to allow actin labelling with phalloidin. CFTR was surface labelled using an anti-HA antibody against the extracellular HA tag. After CFTR labelling, cells were permeabilized and actin was probed using phalloidin-568. CFTR was visible at the cell surface and significantly co-localized with actin (Fig. 3A,B). As a control for our analysis, we rotated the actin image 90°, which significantly reduced the correlation coefficient (Fig. 3B).

### Disruption of the actin cytoskeleton leads to altered A2BR, but not *β*2AR-dependent signaling

**Figure 4:**
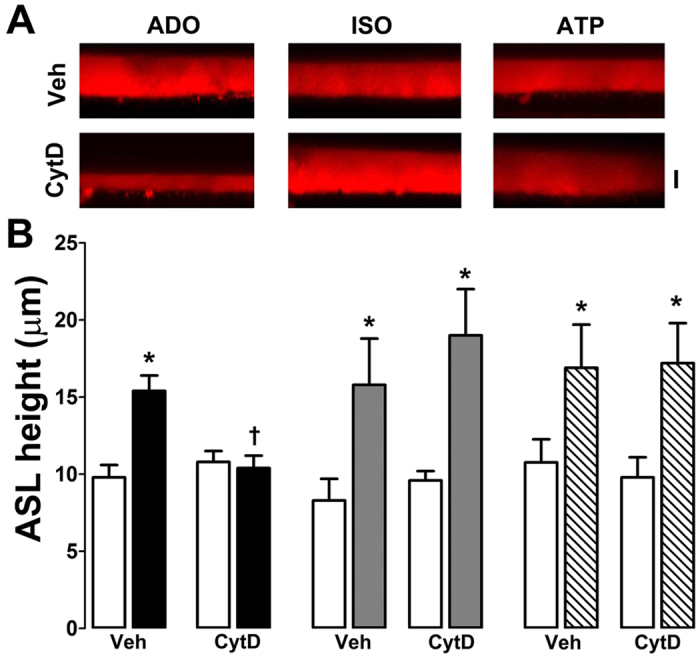
Cytochalasin D abolishes adenosine- but not isoproterenol-mediated ASL secretion in normal HBECs. HBECs were exposed to vehicle (0.1% DMSO) or 10 μM cytochalasin D (CytD) for 30 min before agonist addition. (**A**) Typical XZ confocal micrographs of ASL (red) taken after 10 min exposure to adenosine, isoproterenol and ATP (all at 100 μM) in the presence and absence of 10 μM cytochalasin D in normal HBECs. (**B**) Mean ASL height data. Open bars, control. Closed bars, adenosine; grey bars, isoproterenol; hatched bars, ATP. All n = 6. *p < 0.05 different from vehicle. ^†^p < 0.05 different ± cytochalasin D.

Adenosine-stimulated, CFTR-mediated Cl^−^ secretion is required for ASL hydration^23^. Therefore, to determine whether a similar relationship existed between A2BR/CFTR-mediated fluid secretion and the cytoskeleton in HBECs, we measured ASL height in the presence and absence of cytochalasin D. Actin depolymerization prevented adenosine- but not isoproterenol-stimulated ASL secretion in normal HBECs, suggesting that this effect was specific for adenosine/CFTR-mediated ASL secretion (Fig. 4A,B). As an additional control, we also looked at the effect of actin disruption on ATP/Ca^2+^-activated Cl^−^/ASL secretion and found that cytochalasin D had no effect on this parameter (Fig. 4A,B).

**Figure 5:**
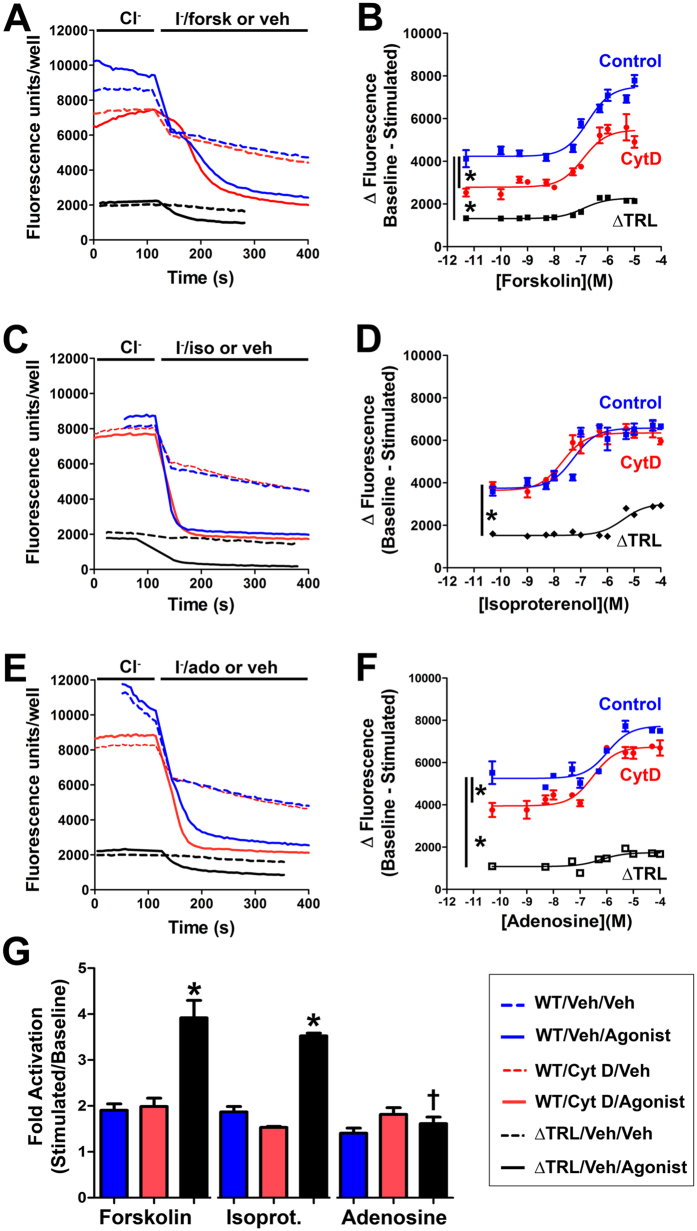
Forskolin and adenosine, but not isoproterenol, elicit cytochalasin D-sensitive CFTR responses. HEK293T cells were cultured in 384-well plates and co-transfected with either wild-type CFTR or CFTR^ΔTRL^ and halide-sensitive YFP. Cells were pretreated for 30 min with 1 μM cytochalasin D or vehicle (0.1% DMSO) in media. Using a multiplate reader, fluorescence was obtained at 0.2 Hz. After baseline readings were obtained, Cl^−^ in the media was exchanged for I^−^ and agonist or vehicle, and the change in fluorescence over time was monitored at 0.2 Hz until a steady state was reached. (**A–C**) Typical traces showing changes in fluorescence for forskolin, isoproterenol and adenosine respectively and their vehicle controls ± cytochalasin D. **(D–F**) Dose response curves for forskolin, isoproterenol and adenosine respectively ± cytochalasin D with wild-type CFTR or CFTR^ΔTRL^ without cytochalasin D. Note, in some cases, error bars were occluded by the graph symbols. (**G**) Bar graph showing the fold-stimulation (maximally stimulated/basal) taken from (**D–F**) for forksolin, isoproterenol and adenosine. Blue bars are control/wild-type CFTR, red bars are cytochalasin D-exposed/wild-type CFTR and black bars show ΔTRL. All data points are n = 12. *denotes significant difference to control or between curves fitted with the equation [Y = Bottom + (Top-Bottom)/(1 + 10^((LogEC_50_ − X)))]. ^†^denotes significant difference to other CFTR^ΔTRL^s.

To test whether CFTR-mediated Cl^−^ secretion was similarly affected in other cell types, we co-expressed wild-type CFTR and halide sensitive YFP in HEK293T cells and measured the degree of YFP-quenching upon exchange of 100 mM Cl^−^ in the bath solution for 100 mM I^−^ as described^24^. In the presence of agonist, exchange of Cl^−^ in the bath solution for I^−^ gave a rapid quenching of YFP fluorescence which was markedly greater when CFTR was co-expressed (Fig. 5A,C,E). Since we cultured cells in 384-well plate format, we were able to generate ~12 point dose responses for all conditions tested and rather than compare individual data points, we curve-fitted each dose response and compared the differences in the curves (Fig. 5B,D,F). Consistent with the data shown in Fig. 4, adenosine- but not isoproterenol- stimulation of CFTR diminished after cytochalasin D exposure over a range of doses (Fig. 5B,D,F). Forskolin-dependent stimulation of CFTR was also cytochalasin D sensitive (Fig. 5A,D). We also co-expressed CFTR^ΔTRL^ with the halide-sensitive YFP and measured its sensitivity to adenosine/isoproterenol. CFTR^ΔTRL^ is typically expressed at lower levels at the plasma membrane and generates smaller currents when stimulated^25^. Similarly, we found that CFTR^ΔTRL^ elicited a smaller change in fluorescence (Fig. 5A–F). However, the relative activation for CFTR^ΔTRL^ was greater than for wild-type CFTR for both forskolin and isoproterenol while the adenosine response was significantly depressed, suggesting that deletion of CFTR’s C-terminus has a greater effect on the ability of adenosine over isoproterenol to stimulate CFTR (Fig. 5G).

**Figure 6:**
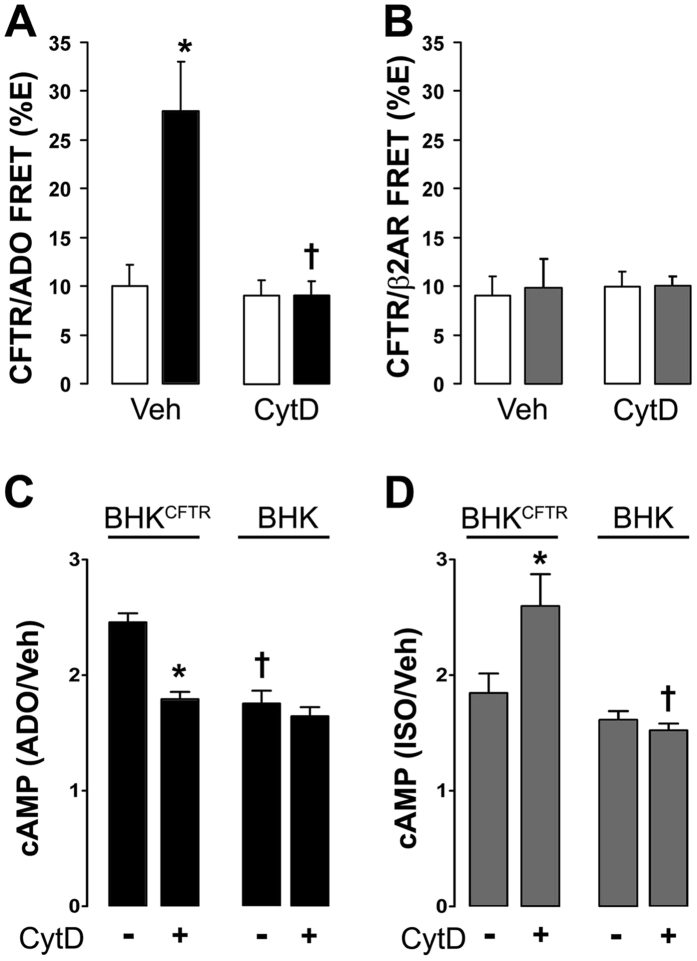
A2BR/CFTR FRET and cAMP production are dependent on an intact actin cytoskeleton. BHK cells were exposed to vehicle (0.1% DMSO) or 10 μM cytochalasin D (CytD). Open bars are controls. Closed bars, adenosine (ADO). Grey bars, isoproterenol (ISO). (**A**) The ADO-induced increase in A2BR*yfp*–CFTR*cfp* FRET was abolished by cytochalasin D (n = 10). (**B**) β_2_AR*yfp*–CFTR*cfp* FRET is not agonist dependent and not affected by cytochalasin D (n = 12). (**C**) Cytochalasin D abolishes CFTR potentiation of ADO-induced cAMP production in BHK^CFTR^ cells and is without effect in BHK cells (n = 12). (**D**) Cytochalasin D had no effect on ISO-induced increases in cAMP levels in BHK^CFTR^ cells, but significantly reduced cAMP production in BHK cells. *p < 0.05 different from vehicle. ^†^p < 0.05 different ± cytochalasin D (n = 12).

We have previously shown that CFTR and A2BR (i) co-immunoprecipitated and underwent adenosine-dependent increases in FRET and (ii) that CFTR expression enhanced adenosine/A2BR-induced increases in cytosolic cAMP^21^. We examined the effect of cytoskeletal disruption on CFTR-A2BR interactions. Pretreatment with the microtubule inhibitor colchicine (10 μM) had no effect on CFTR-A2BR basal or adenosine-induced FRET (12.9 ± 4.6% and 19.6 ± 4.2% respectively; n = 4 and 8). Cytochalasin D had no effect on basal CFTR-A2BR FRET levels, but completely attenuated the adenosine-dependent increase in FRET (Fig. 6A). In contrast, cytochalasin D had no effect on β2AR-CFTR FRET either basally, or post-isoproterenol (Fig. 6B). Cytochalasin D only affected the adenosine-induced increase in cAMP production in the presence of CFTR (Fig. 6C) and was without effect on cAMP levels in BHK cells. While cytochalasin D had no effect on isoproterenol-induced cAMP production in BHK cells, it significantly reduced enhanced agonist-induced cAMP production in BHK^CFTR^ cells (Fig. 6D).

### The C-terminal PDZ domain of A2BR is required for CFTR-dependent cAMP production

**Figure 7:**
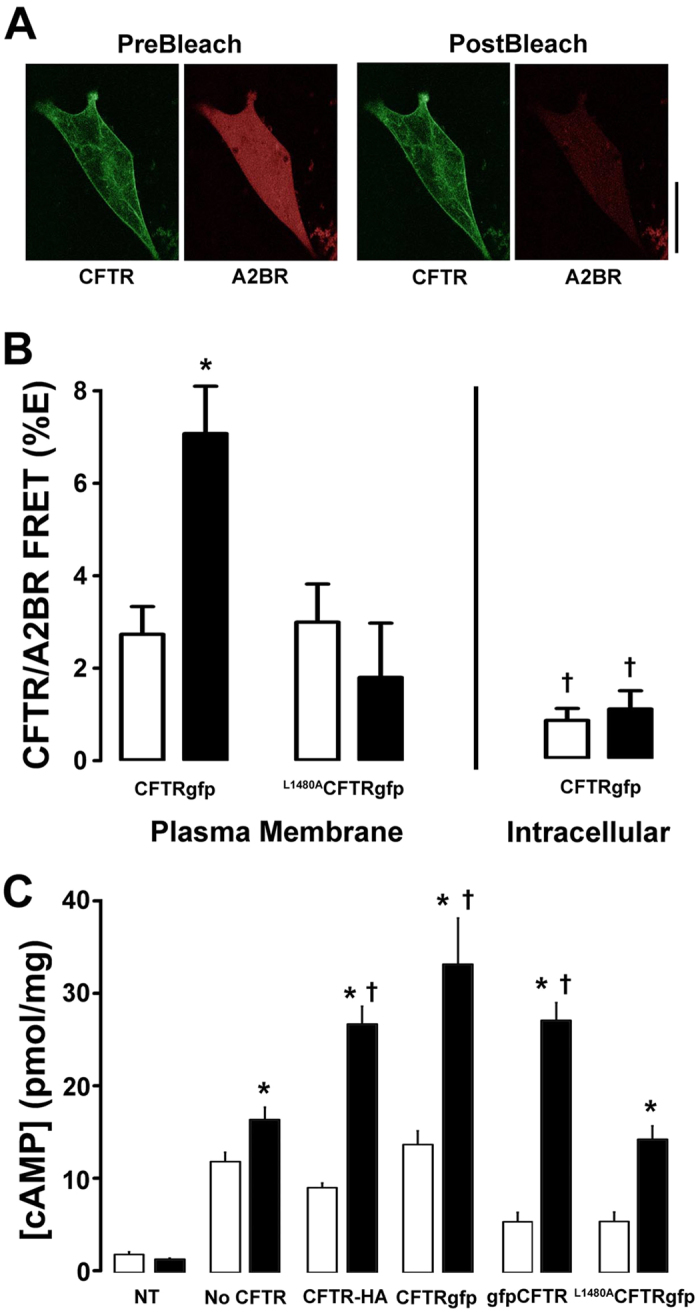
The C-terminus of CFTR is required for CFTR-A2BR interactions at the plasma membrane. (**A**) Typical pre-and post-bleach images obtained by confocal microscopy of gfp-tagged CFTR (green) and mCherry-tagged A2BR (red) expressed in BHK cells. Scale bar is 25 μm. (**B**) Mean FRET efficiency data. *Left*, plasma membrane FRET measured between A2BRmCherry and either wild-type CFTRgfp or the L1480A CFTRgfp mutant under basal or adenosine-stimulated conditions. All n = 7–9. *Right*, intracellular FRET between CFTRgfp and A2BRmCherry ± adenosine. Open bars, vehicle. Closed bars, FRET after 5 min exposure to 10 μM adenosine. (**C**) Intracellular cAMP levels under basal conditions (open bars) and following 5 min adenosine exposure (closed bars) in BHK cells endogenously expressing A2BR and either no CFTR, CFTR tagged with gfp on its C-terminus, extracellularly HA-tagged CFTR and following mutation of the C-terminus (L1480A). All n = 6. NT is non-transfected control. *p < 0.05 different from vehicle. ^†^p < 0.05 different to wild-type or different ± CFTR.

To differentiate between plasma membrane vs. intracellular CFTR/A2BR FRET, we used a CFTRgfp (donor) vs. A2BRmCherry (acceptor) FRET pair and performed acceptor-photobleaching FRET before and 5 min after adenosine exposure. This FRET pair exhibited significant basal FRET in the plasma membrane and underwent similar agonist-dependent FRET at the plasma membrane as in our previous studies with CFTRcfp and A2BRyfp^21^, suggesting that their use was valid (Fig. 7A,B). In contrast, we were unable to detect intracellular FRET between CFTRgfp and A2BRmCherry expressed in BHK cells (Fig. 7B). Using cAMP production as a secondary readout, we observed that CFTRgfp yielded a similar potentiation of adenosine-induced cAMP production as GFP-free CFTR (i.e. HA-CFTR; with a HA tag inserted in its 2^nd^ extracellular loop) and that the placement of the gfp on either the C- or the N- termini did not affect CFTR-dependent cAMP production (Fig. 7). To probe the influence of CFTR’s C- terminus on adenosine-stimulated CFTR/A2BR association and cAMP production, we examined the effect of a CFTR mutant with a disrupted PDZ motif (L1480A)^26^,^27^. ^L1480A^CFTR did not affect basal CFTR/A2BR FRET but abolished the adenosine-induced increase in FRET (Fig. 7B) and significantly reduced CFTR-dependent cAMP production (Fig. 7C), suggesting that CFTR’s C-terminal PDZ motif is critical for the CFTR-dependent enhancement of adenosine signaling.

### Switching A2BR’s and β2AR’s C-termini affects their Interaction with CFTR

**Figure 8:**
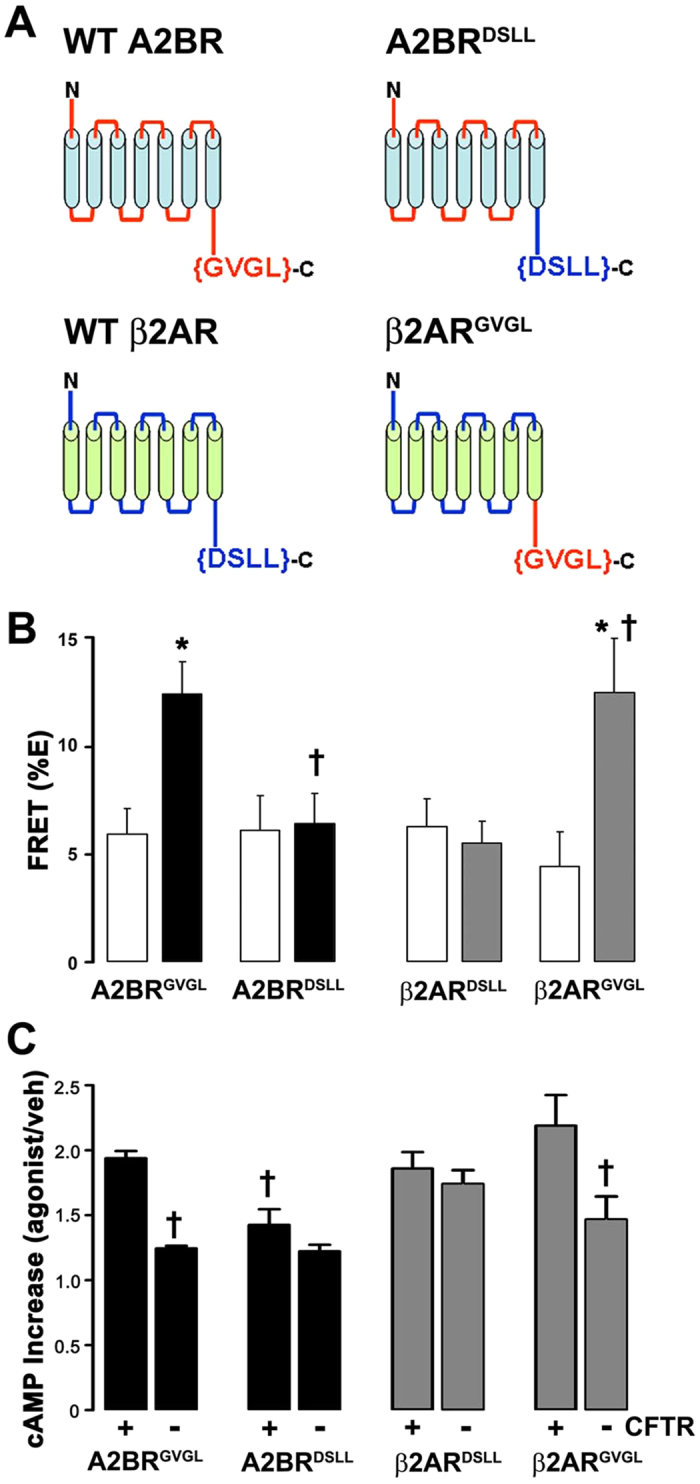
A2BR’s C-terminal PDZ motif confers selective enhancement of GPCR function in the presence of CFTR. (**A**) The last 4 amino acids on A2BR and β2AR are predicted PDZ-binding motifs. The cartoon depicts both wild-type A2BR and β2AR; A2BR where the last 4 amino acids were replaced with those from β2AR (A2BR^DSLL^); and β2AR with the last 4 amino acids from A2BR (β2AR^GVGL^). Mean changes in FRET between CFTR*cfp* and (**B**) wild-type A2BR^GVGL^*yfp or* A2BR^DSLL^*yfp* and (**C**) wild-type β2AR^DSLL^*yfp* and β2AR^GVGL^*yfp* measured in BHK cells. n = 7, 11, 12 and 14 for each pair respectively. Open bars, vehicle; closed bars, ADO; gray bars, ISO. Relative increase in intracellular cAMP levels (agonist/vehicle) for (**D**) A2BR and (**E**) β2AR transfected into BHK^CFTR^ cells as indicated. N = 5, 11, 6 and 12 respectively. *p < 0.05 different from vehicle. ^†^p < 0.05 different to wild-type or different ± CFTR.

Unlike the adenosine-induced cAMP production, isoproterenol/β2AR-induced elevations in cAMP levels are CFTR-independent, and CFTR/β2AR FRET was also unchanged following stimulation with isoproterenol^21^. However, the C-termini of both GPCRs are predicted to contain PDZ-binding motifs. Thus, to test whether the C-termini of A2BR and β2AR affected their interaction with CFTR, we generated new constructs where we swapped the PDZ-binding motifs of these two GPCRs (Fig. 8A). While FRET between CFTRcfp and wild-type A2BRyfp increased upon adenosine stimulation, exchanging the last 4 C-terminal amino acids of A2BR with those from β2AR’s C-terminus abolished the adenosine-induced increase in FRET (i.e. A2BR^GVGL^ to A2BR^DSLL^; Fig. 8B) and also abolished the CFTR-dependent increase in cAMP production (Fig. 8D). In contrast, while β2ARyfp did not increase the %FRET with CFTR after isoproterenol addition, exchange of β2AR’s C-terminus for that of A2BR (i.e. β2AR^DSLL^ to β2AR^GVGL^) conferred an isoproterenol-dependent increase in FRET (Fig. 8C). Note that A2BR^DSLL^ was still functional and increased cAMP levels above baseline (Fig. 8E). However, the increase was of a similar magnitude as β2AR, but less than A2BR (Fig. 8C). In contrast, β2AR^GVGL^–stimulated cAMP production became CFTR-dependent and was significantly greater than that produced by wild-type β2AR (Fig. 8E).

## Discussion

The interaction of CFTR with the actin cytoskeleton has been recognized. For example, actin disruption has been shown to both potentiate and inhibit CFTR activity depending on the experimental conditions^28^,^29^. It has previously been noted that CFTR expression affects actin organization in 16HBE cells^22^. We have found identical results in HEK293T cells and in our primary, well-differentiated HBECs (Fig. 1). We also found that this observation extends not only to actin, but also to ezrin, NHERF1 and NHERF2 and that only cellular localization but not total protein was affected (Fig. 1). Whilst we saw an increased fluorescence intensity near the apical plasma membrane, total protein was not increased (Fig. 1). It is likely that the increased concentration of protein near the apical membrane rather than an increased total amount of protein caused this increase in fluorescence. Similarly, we and others have observed an increase in stromal interacting protein 1 (STIM1) fluorescence when endoplasmic reticulum Ca^2+^ is depleted, which is due to STIM1 aggregation rather than an increase in total STIM1 protein levels^30^,^31^. To further investigate this phenomenon, we looked at the effect of mutating CFTR’s C-terminus on actin and NHERF1 reorganization. Using confocal microscopy, we observed that CFTR’s C-terminal PDZ domain was required to affect F-actin organization and that CFTR with a disrupted PDZ domain (^ΔTRL^CFTR) was no longer able to influence either actin or NHERF1 organization and no longer co-localized with F-actin (Fig. 2A,B). To look at this effect in more detail, we used super resolution microscopy, which surpasses the diffraction limit of light and enables a resolution of ~30 nm^2^. Interestingly, the super resolution analysis revealed (i) discrete CFTR localization at the edge of the cell (e.g. the plasma membrane) that was distinct from intracellular CFTR and (ii) a close correlation between wild-type, but not CFTR^ΔTRL^ and the actin cytoskeleton (Fig. 2C,D). To the best of our knowledge, this is the first time that super resolution microscopy has been used to identify and localize CFTR. Again, using confocal microscopy to analyze surface CFTR, we were able to further highlight the association of CFTR with actin and indeed, it appears that surface CFTR’s expression closely follows that of actin (Fig. 3). Thus, there appears to be a complex interplay between CFTR and the scaffolding proteins that will require further study.

It has previously been shown that latrunculin, which disrupts actin assembly, attenuates CFTR mediated Cl^−^ efflux^22^. Our data indicated that the effects of actin depolymerization on CFTR may also be dependent on how CFTR is activated. That is, cytochalasin D prevented A2BR but not β2AR-dependent increases in ASL height (Fig. 4). However, several other factors can influence Cl^−^ secretion in polarized HBECs. For example, ASL/Cl^−^ secretion occurs under “open circuit conditions”. That is, for secretion to occur, ENaC must first be inhibited to hyperpolarize the apical membrane since the intracellular Cl^−^ concentration is less than the ASL Cl^−^ concentration^32^. Indeed, we have previously shown that adenosine-dependent stimulation of ASL height can be abrogated by activating ENaC^33^. Thus, we cannot exclude the possibility that cytochalasin D is affecting ENaC, or K^+^ channels to prevent ASL secretion. However, the adenosine-dependent decrease in halide-sensitive YFP (Fig. 5), the increase in CFTR-A2BR FRET (Fig. 6A) and the increase in adenosine-stimulated cAMP production (Fig. 6C) were all ablated when the cytoskeleton was disrupted. In contrast, the CFTR-β_2_AR ASL secretion (Fig. 4), FRET (Fig. 6B) and isoproterenol-stimulated cAMP production (Fig. 6D) were cytochalasin D-independent in CFTR-expressing cells. Surprisingly, in the absence of CFTR, β2AR/isoproterenol- cAMP production was cytochalasin D-dependent and enhanced (Fig. 6D), suggesting that GPCR signaling is different in the absence of plasma membrane CFTR. Together, these data highlight the importance of both CFTR and the actin cytoskeleton in regulating A2BR and β2AR signaling and also suggest that these GPCRs signal differently to raise cAMP. The demonstration of similar effects of cytochalasin D in polarized HBECs as in our cell lines (HEK293T and BHK), suggests that our observations are relevant to more complex cell types, such as polarized epithelia. Cytochalasin D causes major cellular disruption. However, we could still generate isoproterenol and ATP-mediated ASL secretion and cAMP production after cytochalasin D exposure, suggesting that this exposure was not toxic and that the HBECs could maintain sufficient polarity and function to generate ASL secretion.

FRET is typically taken as a change in distance between two fluorophores and changes in FRET can be approximately calculated using the equation r = Ro * [(1/E%) − 1]^(1/6)^34^. Thus, with cytochalasin D exposure, we saw an ~15% decrease in FRET between CFTR and the A2BR, which could correspond to a change of ~15 Å. However, changes in FRET can also be caused by changes in orientation, rather than distance of the 2 fluorophores. As such, any FRET data has to be interpreted cautiously. In our opinion, the safest interpretation of the FRET data is that adenosine causes some movement between CFTR and A2BR that is abrogated by cytochalasin D. There was little intracellular FRET and significantly more plasma membrane FRET between CFTR and A2BR, even under basal conditions (Fig. 7). These data suggest that CFTR and A2BR do not associate intracellularly, and only do so when they reach the plasma membrane. It has been suggested that placing GFP on CFTR’s C-terminus affects its PDZ-binding interactions. Using cAMP production as a read out, we compared CFTR gfpCFTR (i.e. N-terminal GFP) or CFTRgfp (i.e. C-terminal GFP). Both constructs still elicited an enhanced adenosine-dependent increase in intracellular cAMP levels (Fig. 7C), suggesting that the CFTR-A2BR interaction was not significantly altered.

PDZ-interactions play a key role in anchoring CFTR, β_2_AR and A2BR to the actin cytoskeleton^12^,^13^. For example, NHERF1 has two PDZ-binding domains that may link CFTR and β_2_AR in macromolecular complexes^13^ and alterations to the C-terminal PDZ-binding motif of CFTR affects its stability in the plasma membrane^35^,^36^,^37^. To test whether CFTR’s C-terminus was important for the CFTR-A2BR interaction, we utilized a mutant CFTR whose C-terminus was altered so as to not bind PDZ domains (^L1480A^CFTRgfp). This mutant has previously been shown to traffic to the plasma membrane and to conduct Cl^− ^27. However, the CFTR-A2BR FRET and the CFTR-dependent increase in cAMP production after adenosine exposure were again significantly diminished (Fig. 7A–C), suggesting that CFTR’s PDZ domain is crucial for this interaction. The role of PDZ binding in enhancing A2BR function was underscored by our GPCR domain swapping experiments (Fig. 7). Both A2BR and β2AR have C-terminal PDZ motifs (Fig. 8A). However, A2BR has a type 2 motif (i.e. X-Φ-X-Φ, where X is any amino acid and Φ is any hydrophobic amino acid) while β2AR has a type 1C-terminal PDZ motif (i.e. X-S/T-X-Φ)^38^. Interestingly, domain swapping did not alter basal levels of FRET for either GPCR and did not fully ablate adenosine-stimulated cAMP production, suggesting that there is a basal level of A2BR-CFTR interaction that is independent of PDZ binding (Fig. 8).

cAMP compartmentalization in polarized epithelia has been well described^39^,^40^. This was initially inferred by the often precise location of key players in the cAMP pathway. For example, PKA is attached to the actin cytoskeleton via NHERF1 and ezrin^41^ and phosphodiesterases that degrade cAMP are also known to be localized sub-apically in several different epithelia including airways^42^. More recently, using genetically encoded, FRET-based cAMP sensors, it has been shown that sub-apical cAMP levels change differentially to cytoplasmic cAMP following stimulation of adenylate cyclase with forskolin, in a CFTR-dependent manner^43^. We have previously shown that CFTR enhances cAMP production by A2BR, but not by β2AR, likely by a CFTR-dependent insertion of A2BR in the plasma membrane, although the mechanism wasn’t fully understood^21^. Our current data suggest that CFTR’s effects on the cytoskeleton were responsible for specifically increasing A2BR-dependent signaling. Naren *et al*. have previously studied the interactions between CFTR, GPCRs and the actin cytoskeleton^20^. Using Cos-7 cells, they found a strong functional PDZ-dependent interaction between β2AR and CFTR, but little actin/PDZ dependency for A2BR. We do not know why we found different results to them. However, Naren *et al*. only tested the interactions at one stimulating dose, whilst we generated full dose responses and compared the curves rather than individual data points (Fig. 5). Alternatively, there may be a species difference between the simian-derived Cos-7 cells used by Naren *et al*. and the human HEK293T cells used in this study (Fig. 5).

In conclusion, we have demonstrated that CFTR’s C-terminus interacts with different GPCRs and also helps shape the actin cytoskeleton. These, and related data, point to an important role for CFTR in cell signaling^21^,^22^, the cells of CF patients with severe mutations likely have altered signaling abilities that are still only being appreciated. Thus, while CFTR/A2BR interactions have been shown to affect cAMP production, ciliary beating and cytokine secretion^21^, the impact of CFTR-dependent cell signaling on CF disease pathogenesis remains to be determined.

## Methods

### Cell Isolation and Culture

Since transfection efficiencies are very low in primary HBECs, we have used HEK293T cells as our main cell line for this study. Where biochemical or imaging experiments necessitated the use of a cell line with high CFTR expression, we used BHK cells that stably express “exotope CFTR”, which has an extracellular HA tag inserted into CFTR’s 3^rd^ extracellular loop^44^. Where transfection was not required, we used normal vs. CF HBECs. Human donor lungs and excised recipient lungs were obtained at the time of transplantation from portions of main stem or lumbar bronchi and cells were harvested by enzymatic digestion in accordance with a protocol that was approved by the UNC Institutional Review Board^45^. Informed consent was obtained from authorized representatives of all organ donors and from all patients. All CF HBECs were obtained from DF508 homozygous patients. Baby Hamster Kidney (BHK) cells were cultured in DMEM/F12 media containing 5% Fetal Bovine Serum (FBS) at 37 °C in 5% CO_2_. Human embryonic kidney (HEK293T) cells were cultured in DMEM media containing 10% Fetal Bovine Serum (FBS) at 37 °C in 5% CO_2_. Both cell types were cultured on glass slides for imaging or 6-well plastic plates for biochemical experiments as described^21^. If required, HEK293T cultures that were ~75% confluent were transfected for ~6 h using Lipofectamine (Invitrogen, Carlsbad, CA, USA) as per the manufacturer’s instructions. The transfection reagents were then removed and the cultures were used for experimentation 24–48 h later.

### Förster resonance energy transfer (FRET)

Acceptor-photobleaching FRET was measured with a TE300 microscope (Nikon Instruments, Melville, NY, USA) using a 60 × 1.2 NA water objective lens, switchable filter wheels (Ludl, Hawthorne, NY, USA) and an Orca CCD camera (Hammamatsu, Bridgewater, NJ, USA) for CFP/YFP FRET pair, or with a SP5 confocal microscope (Leica Microsystems, Buffalo Grove, IL, USA) with a 63 × 1.3 NA glycerol objective lens for the GFP/mCherry FRET pair as previously described^21^,^46^. In brief, the donor (eGFP) was excited at 488 nm and emission collected from 495 nm to 549 nm and the acceptor (mCherry) was excited at 561 nm and emission collected from 580 nm to 654 nm. The acceptor was then photobleached until <5% of the original image intensity remained, then donor and acceptor fluorescence were remeasured. All image analysis was performed using Image J (NIH Freeware). The 4 images in each set (e.g. pre and post bleach for acceptor and donor) were opened as a stack and corresponding regions of interest were drawn around the bleach region in the 4 images. After the image intensities were measured, to determine the FRET efficiency (%E), the following equation was used: ((donorpostbleach-donorprebleach)/donorpostbleach)*100.

### cAMP Measurements

After vehicle or agonist addition, media was aspirated and cells were lysed with 0.1N HCl, centrifuged at 10,000 × g for 10 min and the supernatants were assayed using an enzyme immunoassay kit (Enzo Lifesciences). Pellets were assayed for protein content using the BCA method (Pierce) as described previously^21^.

### Halide-sensitive YFP assay

HEK293T cells were transfected with CFTR and YFP on 10 cm dishes, trypsinized, reseeded in 384 well plates at a density of 10,000 cells per well and assayed 24 h later using the method of Galietta *et al*.^24^. In brief, cells were bathed in PBS^++^ and baseline fluorescence was recorded at 0.2 Hz using a Tecan M1000 multimode plate reader (Tecan) at 37 °C. PBS^++^ was then exchanged for a modified PBS where 100 mM NaCl was replaced with an equal concentration of NaI and agonist or vehicle. Fluorescence was then acquired at 0.2 Hz until a new steady state had been reached. In some cases, cells were incubated with 1 μM Cytochalasin D for 30 minutes prior to starting the assay.

### Western Blotting

Cells were lysed with 1% NP40 buffer on ice. Cell lysates were centrifuged at 10,000 x g for 10 min at 4 °C, and supernatants were collected. Cell lysates (25 μg) were loaded, separated on 3–8% Tris-acetate minigels and transferred to PVDF membrane (Invitrogen) using an iBlot dry transfer system as described^21^,^46^. After the gels were run, they were quartered and each portion, which contained a normal and a CF lane, was separately probed for either actin, ezrin, NHERF1 and NHERF2. All western blots were read using a BioRad Chemidoc system.

### Immunofluorescence

For surface labelling, HA-CFTR, BHK^CFTR^ cultures were blocked for 1 h with 1% BSA and 1% normal goat serum (NGS) at 4 °C in BHK media. After this time, cells were incubated in the same blocking solution with rabbit anti-HA polyclonal antibody (1:300) for an additional 1 h at 4 °C. Intact cells were fixed for 10 min in 4% paraformaldehyde before permeablization with 0.1% Triton-X and staining with Phalloidin-561 (1:1000) as described^44^.

For standard immunofluorescence, HBECs and HEK293T cells were fixed at room temperature with 4% PFA for 30 min and blocked overnight in BSA/NGS before labelling with primary and secondary antibodies and/or phalloidin at room temperature. When changing between antibodies, all cells were washed for 4 × 5 min in PBS. All images were analyzed using Image J by placing regions of interest around the apical membrane to obtain the intensity.

The Pearson Correlation/colocalization plugin in the ImageJ analysis program was used to calculate the co-localization for the signal intensity of the green (CFTR) and red channels (F-actin) as described^47^. Results for the Pearson’s correlation coefficient r describe the linear relationship between the CFTR and F-actin channels. A positive correlation is reflected by the coefficient r value greater than 0 and a negative correlation when the coefficient r value is less than 0.

### Super resolution microscopy

HEK293T cells were fixed in 4% paraformaldehyde before permeabilization with 0.25% Tween20. Cells were then blocked with 10% normal goat serum and 1% BSA in PBS-Tween for 1 h at room temperature and 5 h at 4 °C. The cells were incubated with primary antibodies for rabbit anti-actin (Millipore) and mouse anti-CFTR (UNC, amino acids 1204–1201) at 1:300 dilution at 4 °C overnight. The cells were then incubated with secondary antibodies, phalloidin-488 goat anti-mouse (Life Technologies) and DyLight-633 goat anti-rabbit (Thermo scientific), at a 1:2000 dilution for 1 h in 4 °C. The coverslips were mounted on single concavity slides using 50 mM β-Mercaptoethylamine in PBS, pH 7.4 as the mounting solution, with the edges of the coverslip sealed with Twinsil silicone glue (Leica). Super resolution fluorescence imaging was performed on a Leica SR-GSD-3D localization microcopy using a 160x oil-immersion objective. The image processing package, Leica LAS AF 2.6.1, was used for image acquisition.

### Measurements of airway surface liquid height

PBS (20 μl) containing 2 mg/ml rhodamine-dextran (10 kDa; Life Technologies) was added to cultures at the start of the experiment. In some cases, after addition of PBS, all available fluid was aspirated with a Pasteur pipette to bring ASL volume down to minimal levels. Five predetermined points (one central, four 2 mm from the edge of the culture) were XZ scanned using a confocal microscope (Leica SP5; glycerol 63X immersion lens) as described^23^. Cultures were returned to the incubator between time points. For all studies, ~100 μl perfluorocarbon (PFC) (FC-77; 3 M) was added mucosally during imaging to prevent evaporation of the ASL. As previously described, PFC does not displace ASL and does not affect ASL height, ion transport or mucus transport^48^. ASL heights were then analyzed using ImageJ as described by placing regions of interest around the ASL to determine the height.

### Solutions and Chemicals

Unless noted, all chemicals were purchased from Sigma-Aldrich. The following antibodies were used: Mouse anti-Ezrin (BD Biosciences), rabbit anti-NHERF2 (Sigma-Aldrich), rabbit anti-EBP50 and anti-HA (Abcam), rabbit anti-Actin (Millipore). Secondary antibodies were raised in goat and purchased from Life Technologies and Jackson ImmunoResearch Laboratories. Phalloidin-568 (Life Technologies) was made up at 2 mg/ml in methanol and used at a final concentration of 1 μg/ml. During experiments, cells were maintained in a modified Ringer solution containing (mM): 130 NaCl, 5.1 KCl, 1.2 CaCl_2_, 1.2 MgCl_2_, 10 mM HEPES, 10 glucose, pH 7.4.

### Statistical Methods

All data are presented as mean ± standard error and data was analyzed using Instat3 or Prism (Graphpad). Data were inspected by analysis of variance to test whether or not they were derived from a single population that was normally distributed. Statistical significance between groups was assessed using paired or unpaired *t* test as appropriate. If data were not normally distributed then the Mann-Whitney U test or Wilcoxin matched pairs test were used as appropriate. Values of *n* refer to the number of subjects or the number of cultures used in each group. For HBECs, a minimum of 4 different donors supplied cultures for each experiment. For experiments utilizing HEK293T and BHK cells, all experiments were performed on at least three separate occasions.

## Additional Information

**How to cite this article**: Watson, M. J. *et al*. The Cystic Fibrosis Transmembrane Conductance Regulator (CFTR) Uses its C-Terminus to Regulate the A2B Adenosine Receptor. *Sci. Rep*. **6**, 27390; doi: 10.1038/srep27390 (2016).
